# Dynamics of arable weeds communities in spring and winter wheat under different legume pre-crops during organic conversion

**DOI:** 10.3389/fpls.2026.1769409

**Published:** 2026-03-16

**Authors:** Aušra Arlauskienė, Lina Šarūnaitė, Viktorija Gecaitė, Monika Toleikienė, Žydrė Kadžiulienė

**Affiliations:** Institute of Agriculture, Lithuanian Research Centre for Agriculture and Forestry (LAMMC), Akademija, Lithuania

**Keywords:** legume pre-crops, organic farming, weed abundance and biomass, weed community dynamics, wheat

## Abstract

The transition from conventional to organic farming alters weed communities, their abundance, and competitive interactions with crops. This study assessed changes in annual arable weed communities in winter and spring wheat during the conversion from conventional to organic farming, in relation to different legume preceding crops: peas (P), soybeans (S), a vetch–oat mixture (VOM), and spring barley undersown with red clover (SB+RC). The results showed that during the conversion period, weed species richness, total weed emergence, and the abundance of harmful weed species increased. In the legume cropping years, SB+RC provided the most effective weed suppression, whereas soybeans were the least effective. Weed abundance and biomass were lower in winter wheat than in spring wheat, indicating greater competitive ability of winter wheat. Legume preceding crops generally increased cereal competitiveness and reduced weed biomass, while the effect of SB+RC on weeds differed from other legume treatments. The highest individual weed biomass was recorded for *Galium aparine* and *Fallopia convolvulus*, and by the end of the study the most abundant communities were dominated by *G. aparine* and *Veronica arvensis*. Overall, weed emergence, abundance, and community structure in cereal crops are strongly influenced by crop type, pre-crop effects, environmental conditions, as well as species-specific biological and ecological traits of weeds, underscoring the need for integrated and context-dependent management strategies.

## Introduction

With the intensification of agricultural practices and the expansion of monocultures, plant diversity in arable fields, including weed communities, has declined markedly (Kimmel et al., 2025). The conservation of biodiversity and ecosystem integrity is increasingly recognized as a key condition for ensuring ecosystem resilience and adaptive capacity in the face of increasing agricultural intensification and climate change. Arable weeds are species adapted to continuous anthropogenic disturbance characteristic of intensively cultivated, fertilized, and pesticide-treated agroecosystems (Bourgeois et al., 2019; Munoz et al., 2020). For many arable weed species, cultivated fields represent their primary habitat. In conventional farming systems, long-term chemical weed control promotes the development of resistant populations, reduces species diversity and evenness, and thereby increases the dominance of the most competitive species (Westwood et al., 2018; Mwangi et al., 2024; Boutagayout et al., 2025).

One of the approaches to enhancing biodiversity is organic farming (Madsen et al., 2020; Sanders et al., 2025). However, weed control without chemical inputs remains a major barrier preventing many farmers from transitioning to organic agriculture (Tautges et al., 2016). In the absence of weed control, on average about one third of the potential crop yield may be lost globally due to weed competition compared with ideal weed-free conditions (Gong et al., 2022; Gonzalez-Andujar and Gonzalez-Garcia, 2025).

Weeds not only compete for environmental resources but also increase harvesting costs and reduce product quality (Gage and Schwartz-Lazaro, 2019; Kumar et al., 2021);. Weed management in organic farming is based on practices that, rather than aiming to eradicate weeds, regulate their populations and negative impacts while maintaining biodiversity (MacLaren et al., 2020) and restoring ecosystem services (Kimmel et al., 2025). Organic farming aims to maintain higher weed density, species richness, and evenness, with the goal of weed control being population regulation while accounting for both economic and ecological considerations (Mwangi et al., 2024). In this context, weed management strategies are based on the combined use of several weed suppression tactics that are individually weak but collectively strong (Liebman and Davis, 2009; Kaur et al., 2024). Farming practices have been shown to have a significant impact on weed community composition: crop rotation can reduce weed density by up to 49%, mulching can achieve up to 98% control, and cover crops—depending on species—by 24–85% (Mwangi et al., 2024). Additional measures include competition management as well as direct mechanical or thermal control methods (Westwood et al., 2018). These approaches contribute to reducing the weed seed bank and seedling density and prevent the spread of perennial weeds (Gallandt and Weiner, 2015). In organic arable systems, in contrast to livestock or mixed farming systems, the situation is even more complex, as the potential for internal biological control measures—essential for developing whole-farm systemic resilience—is limited (Wittwer et al., 2023).

Weeds, through their interactions with the entire agroecosystem, also play an important ecological role by supporting biodiversity and agroecosystem functions (MacLaren et al., 2020). Over the past 30 years, many insect and farmland bird populations have declined substantially across Europe and globally and correlational studies link this trend to intensive agricultural practices including widespread pesticide use (Marshall et al., 2003; Quandahor et al., 2024; Reif et al., 2024). It has been suggested that at least 10% weed cover is required to support invertebrate populations (Smith et al., 2020). Effective weed management requires identifying the species composition and ecological diversity of weeds capable of establishing in arable fields. By integrating established and emerging control technologies and building on an understanding of weed biology and ecology, more sustainable weed management and resistance strategies can be developed (Westwood et al., 2018; Schumacher et al., 2024). Although most weed species are frequently mentioned in the literature, information on weed biology is rarely explicitly linked to practical management challenges—an issue that represents a key task for applied weed ecologists (Gallandt, 2014; Chen et al., 2024). Therefore, greater attention should be paid to the traits and processes that determine weed abundance (overall density and biomass), diversity, and community composition (species richness and relative abundance) (MacLaren et al., 2020).

In recent years, weed community assessment has increasingly incorporated ecological diversity metrics alongside traditional measures of density and biomass. Commonly applied indices include the Shannon–Wiener diversity index, species richness measures such as the Margalef index, and evenness (equitability) indices, which allow a more integrative evaluation of species dominance and community structure (Rashmi et al., 2024; Woźniak, 2025). Meta-analytical evidence further indicates that organic farming systems tend to increase weed diversity and evenness compared with conventional systems, highlighting the importance of evaluating both abundance and structural components of weed communities during farming system transitions (Mwangi et al., 2024). Despite numerous studies on organic weed management, multi-year field evidence on how different legume pre-crops influence weed community dynamics during conversion remains limited.

We hypothesized that the transition from chemically intensive farming to organic management would increase weed species diversity and the overall abundance of short-lived weeds (i); and that crop rotation, cover and mixed crops, as well as spring and winter cereals, would differentially affect populations of the harmful weed species *Fallopia convolvulus* (L.) Á. Löve, *Galium aparine* L., *Lamium purpureum* L. and *Veronica arvensis* L. (ii).

The aim of this study was to assess changes in annual weed communities in arable fields under spring and winter wheat during the transition from conventional to organic farming, depending on the type of preceding legume crop.

## Materials and methods

### Experimental site and conditions

The study was conducted from 2019 to 2021 at the Joniškėlis Experimental Station of the Institute of Agriculture, Lithuanian Research Centre for Agriculture and Forestry (LAMMC) (56°21′ N, 24°10′ E), located in the northern part of the Central Lithuanian Lowland, at an elevation of 40–60 m above sea level. The soil of the experimental site is classified as Endocalcari–Endohypogleyic Cambisol (CMg-n-w-can). Its texture is clay loam over silty clay, underlain by sandy loam in deeper layers. At the beginning of the experiment, the topsoil (0–30 cm) was near neutral (pH_KCl_ 6.5), moderately supplied with phosphorus (P_25_ 113 mg kg^-^¹), rich in potassium (K_2_O 267 mg kg^-^¹), moderately humified (organic carbon 22.9 g kg^-^¹), and contained a moderate amount of total nitrogen (N_tot_ 1.13 _g_ kg^-^¹). The bulk density of the plough layer ranged from 1.3 to 1.4 Mg m^-^³, with a total porosity of 40–45% and air-filled porosity of 8–10%. The initial plot size was 15 × 5 m (75 m²), while the experimental plots measured 10 × 2.2 m (22 m²), each replicated four times. The experiment was arranged in two blocks, with plots distributed randomly within each block.

Lithuania is situated within the Nemoral climatic zone, characterized by variable weather conditions — mild, humid summers and cold winters. The mean temperature in January is around –5 °C, while in July it averages 17–18 °C. Annual precipitation typically ranges from 500 to 600 mm, and the growing season lasts approximately 169–202 days. Meteorological data were obtained from the stationary weather station located in Joniškėlis, using temperature and rainfall sensors. No considerable deviations in temperature or precipitation were recorded during the winter season. April was exceptionally dry, with notable rainfall occurring only towards the end of May. In the summer of 2018, precipitation was unevenly distributed — June was relatively dry, whereas July experienced excessive rainfall. The moisture deficit was further intensified by a particularly warm June, when the average daily temperature exceeded the standard climate norm (SCN) by 5.6 °C. In September 2019, excessive moisture complicated winter wheat sowing, as rainfall exceeded the SCN by 12.2 mm. However, a warmer-than-average October promoted good germination and early growth of winter wheat. The winter period was mild, with precipitation levels below the SCN. In spring 2020, rainfall was close to the SCN, except for a dry April. Compared to the SCN, May was cooler by 2.2 °C, while June and July were warmer. These two months were also marked by excessive rainfall — 46.5 mm and 38.8 mm above the SCN, respectively. Overall, the 2020 growing season showed an uneven rainfall pattern: insufficient moisture in the first half of the season and surplus rainfall in the second half. October and November were both warmer and wetter than the SCN. Precipitation in January 2021 did not compensate for the deficit observed in February, indicating that the winter of 2021 was relatively dry. Conversely, spring was cool and humid. The growing season featured heavy rainfall in May (92.2 mm above the SCN) and unusually hot conditions in June and July, with temperatures exceeding the SCN by 7.5 °C and 6.8 °C, respectively. August was characterized by intense rainfall — more than double the SCN.

### Experimental design and details

The research was carried out at the Joniškėlis Experimental Station. Prior to the establishment of the experiment, conventional tillage practices were applied, and both pesticides and mineral fertilizers were used. Winter and spring cereals accounted for approximately 75–80% of the cropping structure, while the remaining part consisted of field peas and winter oilseed rape.

The field experiment was arranged in the following crop rotation sequence: 2019 – barley (*Hordeum vulgare* L.) and legumes → 2020 – winter wheat (*Triticum aestivum* L.) → 2021 – spring wheat (*Triticum aestivum* L.). The crop species and cultivation practices are presented in **Table 1**. In the year of experiment establishment (2019), the following crops were sown according to the experimental design: spring barley (cv. *Ūla*), spring barley undersown with red clover (*Trifolium pratense* L., cv. *Vyliai*), field pea (*Pisum sativum* L., cv. *Respect*), soybean (*Glycine max* (L.) Merr., cv. *Merlin*), and a mixture of common vetch (*Vicia sativa* L., cv. *Aisiai*) and oats (*Avena sativa* L., cv. *Migla DS*).Sowing rates were as follows: 400 seeds m^-^² for spring barley, 150 seeds m^-^² for red clover (undersown), 100 seeds m^-^² for field pea, 60 seeds m^-^² for soybean, and for the vetch–oat mixture — 50 and 200 seeds m^-^², respectively. Cereals (spring barley, spring and winter wheat, peas, and a vetch–oat mixture) were sown using a seeder with disc harrows at 12.5 cm spacing. Soybean was sown with a seeder at 25 cm spacing. For the red clover undersowing, a seeder with tine harrows was used to ensure uniform seed distribution and optimal plant density.

**Table 1:** Crops and dates of main technological operations.

Year	Technological operation	Date
2019	Pre-sowing soil preparation by double cultivation	24–25 Apr 2019
	Sowing of spring barley (SB), pea (P), and mixture of vetch and oats (VOM)	25 Apr 2019
	Sowing of soybean (S)	10 May 2019
	Undersowing red clover in barley (SB+RC)	22 May 2019
	Harvesting of spring barley and pea	5 Aug 2019
	Harvesting of vetch and oat mixture	29 Aug 2019
	Harvesting of soybean	16 Sep 2019
	Plant residues (stubble, straw, red clover biomass) were incorporated using a disc cultivator to a 10 cm depth	15 Sep 2019
	Ploughing to a 25 cm depth	23 Sep 2019
	Soil cultivation and winter wheat sowing	29 Sep 2019
2020	Harvesting of winter wheat (WW)	27 Jul 2020
	Plant residues (stubble, straw) were incorporated using a disc cultivator to a 10 cm depth	29 Jul 2020
	Ploughing to a 25 cm depth	30 Sep 2020
2021	Pre-sowing soil preparation by double cultivation	22–23 Apr 2021
	Sowing of spring wheat (SW)	23 Apr 2021
	Harvesting of spring wheat	16 Aug 2021

In the first year, weed infestation (number of weeds, units m^-2^) in summer cereals (BBCH 30) and legumes (BBCH 35) was determined by taking four samples from each plot of 50 x 50 cm using a wire frame. Weeds were classified into species according to biological and agronomic classification. In winter and spring wheat, weed infestation was assessed twice in each plot in four 50 x 50 cm sites: the first time during the tillering stage (BBCH 30), the number of weeds (units m²) and species composition were determined. The weed infestation was assessed for the second time during grain development (stage BBCH 78) by determining their number, species composition, and mass (g m^-2^). The dry matter mass of weeds was determined by drying and weighing them. Each weed species was weighed separately, and the mass of a single weed was calculated. The following weed species were identified during the study: *Capsella bursa-pastoris* (L.) Medik., *Chenopodium album* L., *Cirsium arvense* (L.) Scop., *Consolida regalis* Gray, *Euphorbia helioscopia* L., *Fallopia convolvulus* (L.) Á. Löve, *Fumaria officinalis* L.*, Galium aparine* L., *Lamium purpureum* L., *Myosotis arvensis* (L.) Hill, *Polygonum aviculare* L., *Polygonum lapathifolium* L., *Sinapis arvensis* L., *Stachys palustris* L., *Stellaria media* (L.) Vill, *Sonchus arvensis* L., *Taraxacum officinale* F. H. Wigg, *Thlaspi arvense* L., *Tripleurospermum perforatum* (Merat) M. Laínz, *Veronica arvensis* L., *Viola arvensis* Murray. It was found that the most widespread short-lived dicotyledonous weeds were *F. convolvulus*, *G. aparine*, *L. purpureum* and *V. arvensis*. The harmfulness of weeds was assessed based on the Lithuanian publication “Weeds: Catalogue” (Špokienė and Povilionienė, 2003).

### Methods of statistical data analysis

Weed plant data were analyzed using one-way analysis of variance (ANOVA). The factor used for statistical analysis was the legume crop. Significant differences were assessed with an F-test at the p < 0.05 and p < 0.01 levels. *Post hoc* comparisons were carried out using Tukey’s test at p < 0.05, where means sharing the same letter are not significantly different. Statistical analyses were conducted using Statistica software, version 7.1 (StatSoft Inc., Tulsa, OK, USA).

## Results

### Number of species

In the year the experiment was set up (2019), the number of weed species ranged from 6.0 to 8.0 units m^-2^, and by the end of the study (2021), it had increased to 6.8–10.3 units m^-2^ (**Table 2**). The following short-lived weed species, often found on chemical farms, dominated in legume crops: *F. convolvulus, G. aparine, L. purpureum, V. arvensis.* These weeds accounted for an average of 67.9% of all short-lived weeds. When red clover was undersown in spring barley (SB+RC), the number of weed species was significantly lower than in the pea crop (**Table 2**). In the second year (2020), there were no significant differences between the treatments. In the third year of the study, the increase in the number of weed species was due to preceding legume crops (except peas).

**Table 2:** Variation in the number of annual weed species, units m^-2^.

Crop sequence	2019	2020 WW	2021 SW	Change %	Mean
SB-WW-SW	7.8abc	10.0b	10.0bc	+28.2	9.3d
SB+RC-WW-SW	6.0a	9.0ab	8.0abc	+33.3	7.7a
P-WW-SW	8.0c	9.0ab	6.8a	-15.0	7.9ab
S-WW-SW	7.3abc	9.7ab	10.3c	+41.1	9.1cd
VOM-WW-SW	7.3abc	9.7ab	9.8bc	+34.2	8.9bcd
Mean	7.3A	9.5C	9.0C	+23.3	8.6

SB, spring barley; WW, winter wheat; SW, spring wheat; RC, red clover; P, pea; S, soya; VOM, vetch and oats mixture. Data with different letters within individual interactions are significantly different at the *p* < 0.05 level; Capital letters indicate significant differences between years (p < 0.05).

During the study period, the number of weed species increased by an average of 23.3%. The greatest increase in weed diversity was observed in the crop rotation cycle with soybeans.

### Number of weeds in legume crops

One-way ANOVA indicated significant effects of legume on harmful weed abundance (p < 0.05). *Post hoc* Tukey tests identified which treatments differed significantly. In terms of species composition, there was significantly more *F. convolvulus* and significantly less *V. arvensis* per square meter (in average) *(***Table 3**). A more pronounced influence of leguminous plants was observed only in the change in the number of *G. aparine*. These weeds were most abundant in the later-emerging soybean crop, and least abundant in the vetch-oats mixture and in the barley with undersown RC. These plants are likely to have effectively shielded the soil surface from light. On average, *F. convolvulus* was 22.9% lower in legume crops than in barley. Emergence of *L. purpureum* and *V. arvensis* tended to increase in legume crops compared to barley. The highest number of harmful weeds was observed in the soybean crop. Compared to soybean, the number of harmful weeds was generally lower in the spring barley crop with undersown RC. The total number of short-lived weeds did not differ significantly between crops. Compared to barley, the legume crops reduced the emergence of *F. convolvulus* and *G. aparine*.

**Table 3:** Emergence of annual and major harmful weeds in legume crops, 2019.

Legume crops	Harmful annual weeds, units m^-2^					Total annual weeds, units m^-2^
	*F. convolvulus*	*G. aparine*	*L. purpureum*	*V. arvensis*	Total	
SB	17b	12abc	9ab	2ab	41abc	65b
SB+RC	14ab	8abc	8ab	2ab	31a	51ab
P	12ab	8abc	14b	5ab	39abc	59ab
S	13ab	15c	13ab	6b	47c	54ab
VOM	15ab	7a	14ab	4ab	40ac	62ab
Weed sp.	14D	10.0B	12BCD	4A	39	58

SB, spring barley; RC, red clover; P, pea; S, soya; VOM, vetch and oats mixture. Data with different letters within individual interactions are significantly different at the *p* < 0.05 level; Capital letters indicate significant differences between weed species (p < 0.05).

### Influence of preceding crops on weed numbers in cereal crops

One-way analysis of variance indicated a significant effect of legume pre-crops on total weed abundance (p < 0.05), harmful weed abundance (p < 0.01), and the abundance of *G. aparine* (p < 0.05), *L. purpureum* (p < 0.01), and *V. arvensis* (p < 0.05) in winter wheat. In the winter wheat crop grown after legume pre-crops, *F. convolvulus* was generally more (in average) abundant than other weed species (**Table 4**). Higher emergence of these weeds was observed after the soybean and vetch-oats mixture compared to the pea pre-crop. The highest emergence of *G*. *aparine* was due to the vetch-oats mixture pre-crop, while the lowest emergence was due to spring barley and pea. *L. purpureum* emerged most abundantly in winter wheat grown after barley with undersow RC. Legume pre-crops (except peas) resulted in the highest number of short-lived harmful weeds. The total number of short-lived weeds followed a similar pattern.

**Table 4:** Total emergence of annual and major harmful weeds in two years of wheat cultivation.

Pre-crops	Harmful annuals weeds, units m^-2^					Total annual weeds, units m^-2^
	*F. convolvulus*	*G. aparine*	*L. purpureum*	*V. arvensis*	Total	
In winter wheat 2020						
SB-WW	24 def	12 ab	12 ab	11 abc	60 ab	84 abc
SB+RC-WW	25 def	8 a	25 d	22 abc	80 d	101 bc
P-WW	19 cd	9 a	15 ab	5 a	48 a	68 a
S-WW	29 f	12 ab	15 ab	22 abc	78 bcd	111 bc
VOM-WW	28 ef	19 bcde	15 ab	15 abc	77 bcd	114 c
Mean	25C	12A	17B	15AB	69A	96A
In spring wheat 2021						
+						
SB-WW-SW	13 bc	13 ab	9 a	17 abc	52 ab	95 a
SB+RC-WW-SW	7 ab	21 cde	8 a	27 c	63 ab	126 c
P-WW-SW	3 a	19 bcde	12 ab	22 abc	56 ab	113 abc
S-WW-SW	8 ab	25 e	18 bcd	16 abc	67 b	92 a
VOM-WW-SW	7 ab	14 abc	10 ab	18 abc	49ab	103 abc
Mean	8A	18BCD	12AB	20D	57A	106A

SB, spring barley; WW, winter wheat; SW, spring wheat; RC, red clover; P, pea; S, soya; VOM, vetch and oats mixture. Data with different letters within individual interactions are significantly different at the *p* < 0.05 level; Capital letters indicate significant differences between weed species (p < 0.05).

In the second year after the legume pre-crops, the timing of tillage and sowing of spring wheat was shifted, which reduced the number of harmful weeds. ANOVA indicated that legume pre-crops significantly affected the total number of annual weeds (p < 0.01), as well as the abundance of *F. convolvulus* (p < 0.05) and *G. aparine* (p < 0.05) in spring wheat. Compared to winter wheat, spring wheat had significantly lower emergence of *F. convolvulus* and *L. purpureum* and higher emergence of *G. aparine*. The number of *V*. *arvensis* increased consistently from year to year and did not vary significantly and was independent of legume pre-crops. As regards the emergence of the harmful weeds, it was found that all the legume pre-crops (with the exception of the vetch-oats mixture) led to a higher emergence rate compared to barley. *F. convolvulus* emerged least when wheat was grown after peas, with a significant difference compared to the spring barley pre-crop. *G. aparine* emerged significantly more in spring wheat grown after barley with undersown RC and soybeans. *L*. *purpureum* was most abundant in spring wheat after soybeans. The highest total number of weeds emerged in wheat after spring barley with undersown RC and the lowest one was determined after soybeans.

Compared to the start of the experiment, the number of total and harmful weeds increased by 46.2 and 82.8% respectively. However, the proportion of harmful weeds in the total number of weeds was 53.8%, compared to 67.2% at the beginning of the experiment.

### Variation in weed number and mass during the cereal growing season (weed suppression ability of wheat)

**Figure 1:**
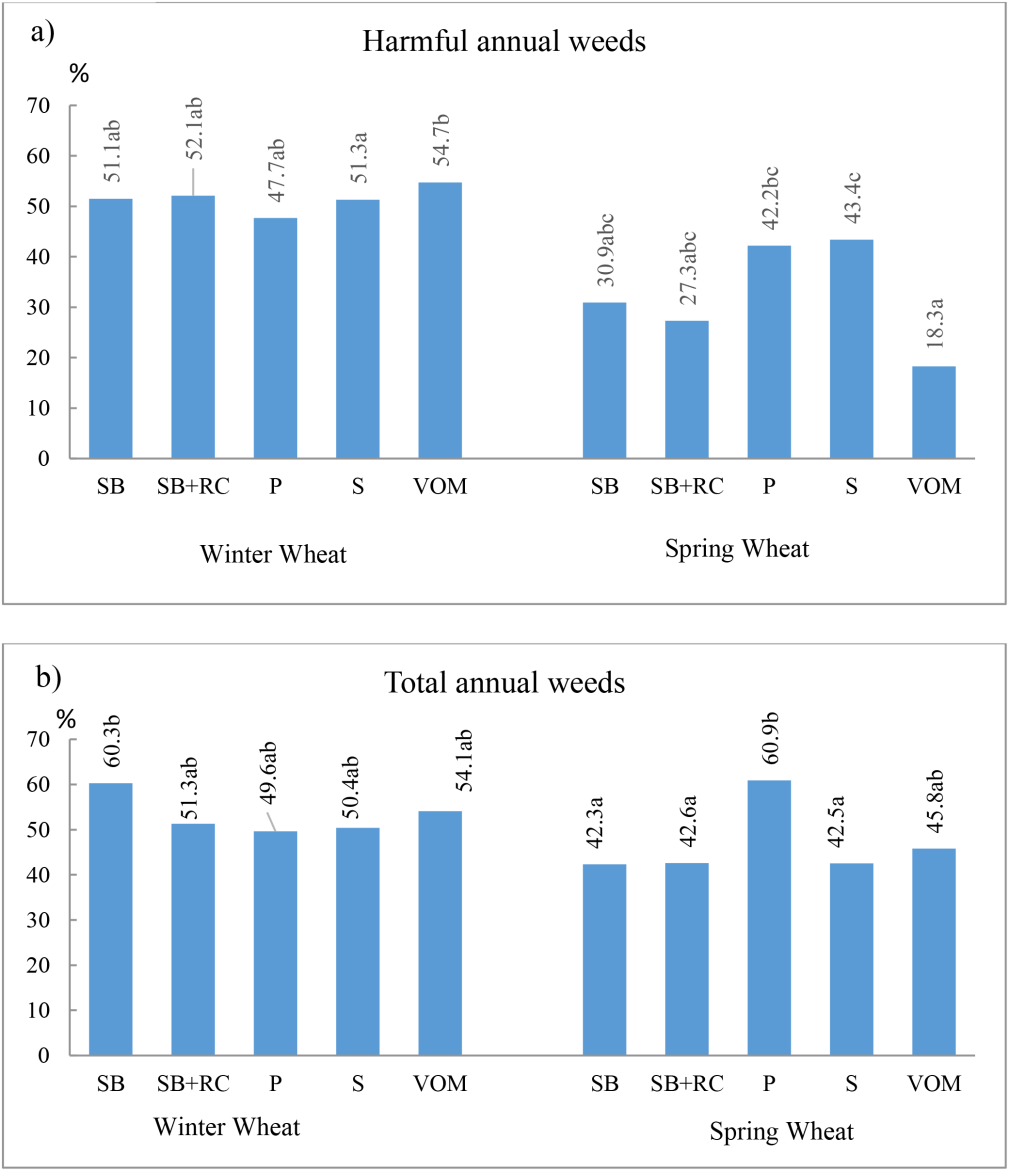
Variation in the number of weeds during the growing season (BBCH 30–78) in winter wheat (2020) and spring wheat (2021). **(a)** Harmful annual weeds. **(b)** Total annual weeds. SB – spring barley; WW – winter wheat; SW – spring wheat; RC – red clover; P – pea; S – soybean; VOM – vetch and oats mixture. Data with different letters indicate significant differences at *p* < 0.05.

**Figure 1** shows the variation (in percentage) of the number of harmful short-lived weeds (total) and the total number of weeds during the growing season (from BBCH30 to BBCH78). During this period, in winter wheat, the number of short-lived harmful weeds decreased from 47.7 to 54.7% and did not depend significantly on the legume pre-crop (**Figure 1a**).

Winter wheat suppressed *L. purpureum* and *V. arvensi*s fairly well (especially after P). Spring wheat was less competitive and reduced the number of weeds during the growing season by 18.3–43.4% compared to the level at the beginning of the growing season. Here the influence of the pre-crop was more pronounced. In spring wheat, the most reduction of short-lived harmful weeds during the growing season was achieved by the S pre-crop and the least – by VOM. There were significant differences between these data. Weed species behaved differently. The number of late-emerging *F. convolvulus* even increased during the growing season, especially after P and S pre-crops. *V*. *arvensis* showed a marked decrease in numbers after S and VOM pre-crops, whereas it tended to increase after other pre-crops. During the growing season, the most significant decrease was observed in the number of relatively early maturing *L. purpureum*, especially after P and S pre-crops. During the winter wheat growing season, the total number of short-lived weeds decreased by between 49.6 and 60.3% and was not dependent on legume pre-crops (**Figure 1b**). In spring wheat, this indicator was 6.3% lower on average. The total number of weeds was reduced by P pre-crop, which showed a significant difference compared to other pre-crops (except VOM). In spring wheat, legumes had a significant effect on the reduction of total and harmful weed numbers during the growing season (p < 0.01 and p < 0.05, respectively) and mass (p < 0.01).

**Figure 2:**
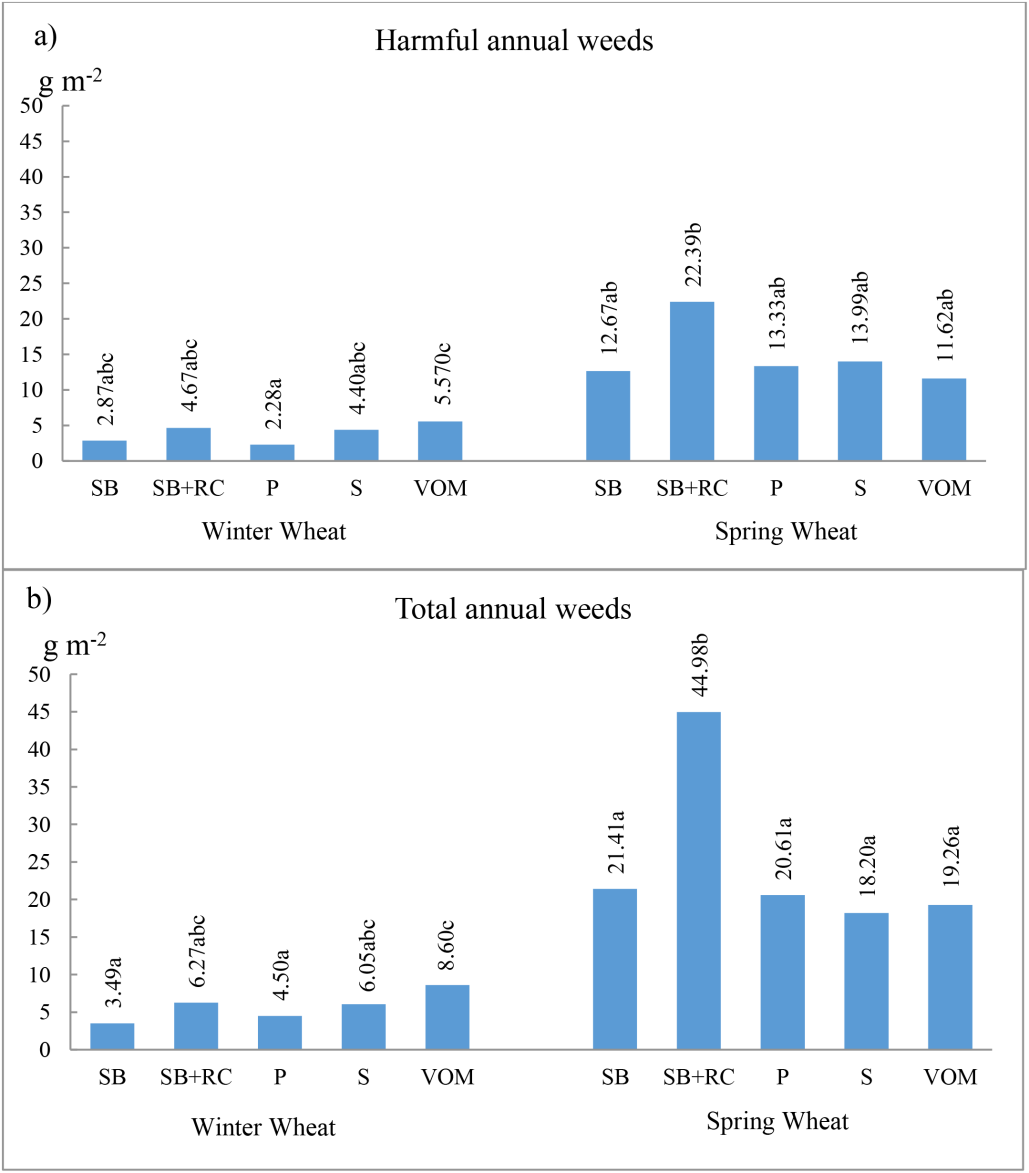
Air-dry mass of weeds before wheat harvesting. **(a)** Air-dry mass of harmful annual weeds. **(b)** Air-dry mass of total annual weeds. SB – spring barley; WW – winter wheat; SW – spring wheat; RC – red clover; P – pea; S – soybean; VOM – vetch and oats mixture. Data with different letters within individual interactions are significantly different at *p* < 0.05.

The most short-lived harmful weeds accounted for 68.3 and 59.4% of the total short-lived weed mass (**Figures 2a, b**). The influence of pre-crops on both the mass of the harmful and total weeds was similar. On average, the highest total and short-lived harmful weed masses were found in the spring wheat crop. In winter wheat, the mass of short-lived weeds was not significantly influenced by the pre-crop. The spring wheat crop had on average 4.3 times higher weed mass than the winter wheat crop. The total and the air-dry mass of harmful weeds was significantly increased by SB+RC pre-crop.

### Harmfulness of weeds

**Figure 3:**
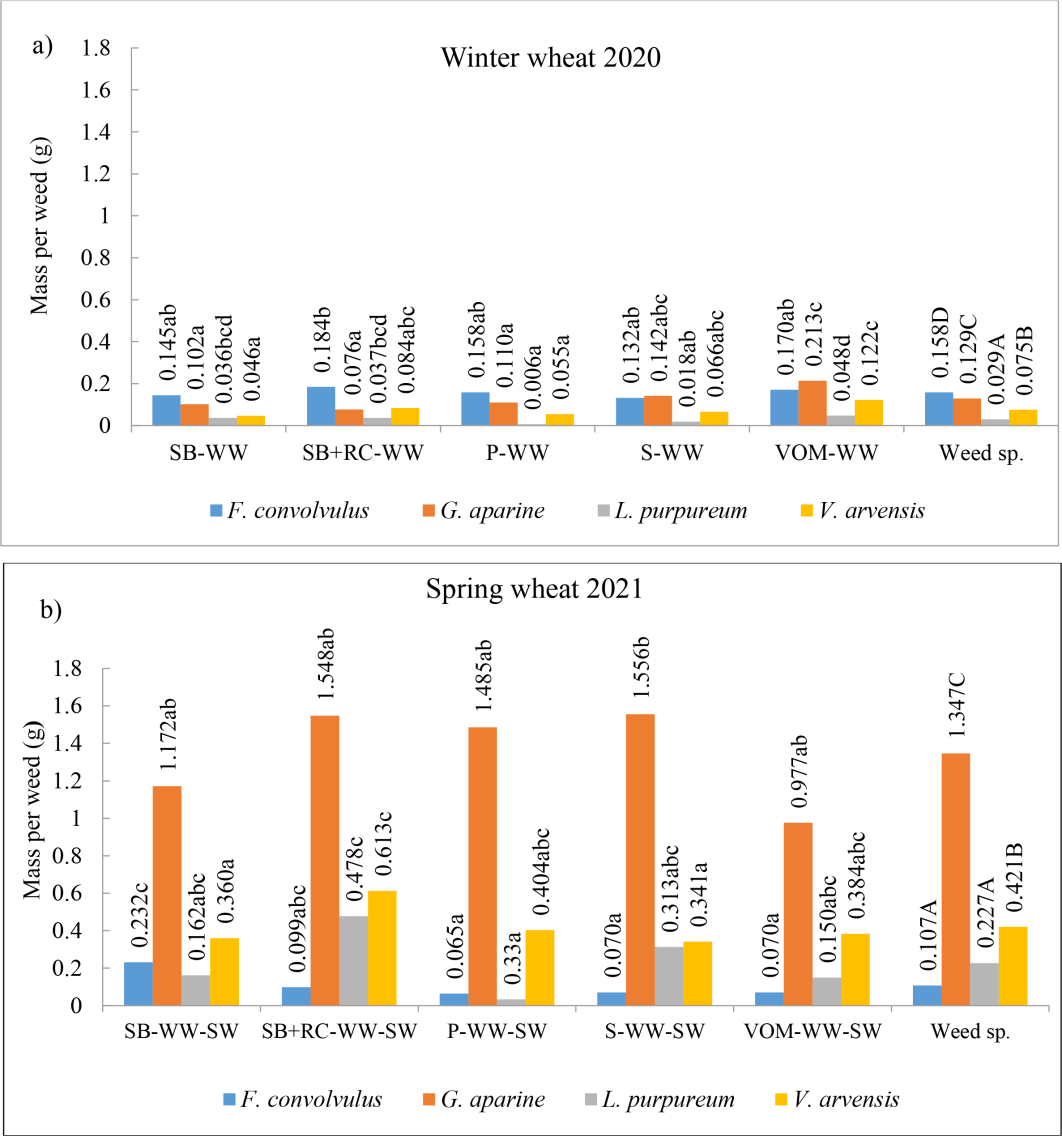
Biomass per plant of annual harmful weeds in winter wheat **(a)** and spring wheat **(b)**. SB, spring barley; WW, winter wheat; SW, spring wheat; RC, red clover; P; pea; S; soya; VOM, vetch and oats mixture. Data with different letters within individual interactions are significantly different at the *p* < 0.05 level; Capital letters indicate significant differences between weed species (p < 0.05).

One-way analysis of variance (ANOVA) indicated a significant effect of legume pre-crops on the individual plant biomass of *G. aparine* (p < 0.05) and *L. purpureum* (p < 0.05) in winter wheat. The weed *F. convolvulus* had a significant effect on total weed biomass under the influence of legume pre-crops in both winter and spring wheat (p < 0.05). Comparison of the air-dry mass of short-lived harmful weed species showed that *F. convolvulus* produced the highest mass per weed in winter wheat. This weeds species accounted for the significantly highest proportion (44.8% on average) of the total weed mass (**Figure 3**, **Table 5**). The mass of *F. convolvulus* per weed was not significantly affected by the pre-crop, but the highest proportion of the mass of this weed was observed in winter wheat grown after SB, SB+RC and S. After the legume pre-crop in the second year of spring wheat, the mass of *F. convolvulus* per weed and the air-dry mass proportion in the total mass of total weeds were reduced significantly. The highest mass of this weed was in wheat grown after SB. On average, the mass per *G. aparine* weed and its proportion in the total mass of weeds was generally lower than for *F. convolvulus* and higher than for *L. purpureum* and *V. arvensis*. The mass of these weeds was not influenced by legume pre-crops. However, in spring wheat, the mass of one *G. aparine* weed increased 10.4-fold compared to winter wheat. The mass of these weeds also accounted for the highest proportion in the mass of total weeds (32.8% on average). The highest mass per weed was in the soybeans sequence and the lowest one was in the vetch and oats mixture. The highest proportion of *G. aparine* in the total weed mass was observed when spring wheat was grown in a sequence after legumes (pea and soybeans).

**Table 5:** Share of annual harmful weeds in total weed biomassin.

Pre-crops	Share of annual harmful weeds, %							
	*F. convol-vulus*	*G. aparine*	*L. Purpur-eum*	*V. arvensis*	*F. convol-vulus*	*G. aparine*	*L. Purpur-eum*	*V. arvensis*
Winter wheat 2020					Spring wheat 2021			
SB-WW	56.0 c	13.7 abc	3.9 d	8.0 ab	18.4 b	16.8 a	2.6 b	19.6 ab
SB+RC-WW	51.6 bc	9.0 abc	2.9 bcd	9.6 ba	5.6 a	29.0 abc	1.4 ab	14.6 ab
P-WW	34.3 a	7.2 a	0.4 a	14.7 b	4.2 a	40.3 bc	0.3 ab	19.3 ab
S-WW	46.8 abc	16.2 c	1.2 ab	9.8 ab	10.1 ab	49.9 c	2.5 ab	13.1 ab
VOM-WW	35.3 a	14.4 abc	2.1 abcd	11.3 ab	8.4 ab	28.3 abc	1.4 ab	23.0 b
Weed sp.	44.8C	12.1B	2.1A	10.7B	9.3B	32.8D	1.6A	17.9C

SB, spring barley; WW, winter wheat; SW, spring wheat; RC, red clover; P; pea; S; soya; VOM, vetch and oats mixture. Data with different letters within individual interactions are significantly different at the *p* < 0.05 level; Capital letters indicate significant differences between weed species (p < 0.05).

In both winter and spring wheat, *L. purpureum* had the lowest mass per weed and accounted for a very small proportion of the total weed mass. A more pronounced influence of the pre-crops was found only in the second year of wheat cultivation. Compared to the control, the mass of *L. purpureum* per weed increased in the cereal rotations with legumes (SB+RC, P, S).

*V. arvensis* had a fairly stable position, with moderate values for the data per weed mass. In winter and spring wheat, they accounted for 10.7 and 17.9% of the total weed air-dry mass, respectively. In spring wheat, *V. arvensis* had significantly higher mass per weed than in winter wheat. The mass per weed was significantly increased by SB+RC pre-crop compared to the control.

## Discussion

Organic farming systems generally promote higher weed species diversity and, in many cases, greater weed abundance compared with conventional agriculture (Mwangi et al., 2024). Madsen et al. (2020) reported that organic systems incorporating cover crops reduce the soil weed seed bank while increasing plant diversity. The long-term dynamics of weed infestation largely depend on the ability to prevent weed seeds from entering and replenishing the soil seed bank (Gallandt, 2014; Sinkevičienė et al., 2025). This is consistent with multi-year seedbank studies demonstrating that crop rotational sequence selection exerts strong legacy effects on weed seedbank density and composition, with competitive cereal-dominated rotations leading to progressive depletion of viable weed propagules (Gurusinghe et al., 2022).

In our study, during the third year of the transition from conventional to organic farming, both weed species richness and the abundance of harmful species, as well as total weed density, increased. Gong et al. (2022) indicated that organic farming can enhance biodiversity by up to 23%; however, yield reductions of a similar magnitude may also occur. Such patterns likely reflect the temporary release from chemical control combined with rotational restructuring and environmental filtering processes during the conversion period. Weed emergence dynamics during conversion are strongly shaped by environmental cues and soil disturbance timing, which regulate seed dormancy release and synchrony of emergence flushes (Travlos et al., 2020; Oreja et al., 2023.

### Legume crops in crop rotation

In organic farming systems, the integration of legume crops into crop rotations is essential for nitrogen accumulation and the maintenance of soil fertility (Barbieri et al., 2023). However, legumes often emerge more slowly and may initially exert weaker competitive pressure on weeds. Recent evidence suggests that the effect of legumes on weed dynamics depends less on their presence per se and more on their position within the rotational sequence and the competitiveness of preceding and subsequent crops (Gurusinghe et al., 2022). Legumes generally require a larger growing space and are therefore sown at wider row spacings. They often emerge more slowly and cover the soil surface later, resulting in weaker competition with weeds, as noted in previous studies (Savić et al., 2025; (Gonzalez-Andujar and Gonzalez-Garcia, 2025) and observed, for example, in semi-leafless pea cultivars (Jacob et al., 2016). Compared with other legumes, soybeans are sown and harvested later, which allows additional pre-sowing tillage for weed control. Nevertheless, soybeans are particularly sensitive to weed competition during early growth stages, and yield losses depend on the intensity and duration of competition, environmental conditions, and applied management practices (Soltani et al., 2017; Toleikiene et al., 2021). In our research, the variety and seed rate for peas were appropriately selected.

Legume crops provide less favorable conditions for weeds that require physical support, such as *G. aparine*. Håkansson (1983) demonstrated that climbing or twining weeds (*F. convolvulus*, *G. aparine*) perform poorly under higher crop stand densities. In contrast, spring- emerging species such as *L. purpureum* and *V. arvensis* may be more competitive under these conditions; their ecological importance in agroecosystems was highlighted by Smit et al. (2020).

A one-year assessment of weed infestation does not necessarily reflect the long-term benefits of weed control, although some studies indicate that a substantial reduction in weed seed production can have lasting effects. Within crop rotations, weeds are continuously exposed to multiple disturbances, including soil tillage, sowing time and crop structure, crop competition, harvest timing, and post-harvest management practices (Woźniak, 2025). The greater the diversity of the crop rotation, the more effectively weeds can be controlled at different stages of their life cycle (Arlauskienė et al., 2021; Woźniak, 2025). Recent long-term analyses of cereal–legume rotations further demonstrate that weed community trajectories shift systematically over time depending on rotational structure and management intensity (Gonzalez-Andujar and Gonzalez-Garcia, 2025). Anderson (2010) recommends alternating winter and spring crops every two years to exploit the half-lives of weed seeds in the soil or to modify soil disturbance effects on weed community dynamics and long-term population trajectories (Hernandez-Plaza et al., 2015; MacLaren et al., 2020).

Weed germination at the beginning of the growing season may have been influenced by the accumulated seed bank in the top layer of the soil (0–5 cm), conditions suitable for germination (light, moisture and heat), and by plant residues, whose intermediate decomposition products stimulate or inhibit the germination of the seeds (allelopathic effect) (Gallandt, 2014; Kumar et al., 2021).

In our study, during the third year (2021), the highest weed emergence occurred in crop sequences including legumes, except for soybean. In spring wheat, the greatest weed biomass was recorded following the SB+RC pre-crop, whereas the lowest biomass was observed after grain legumes (P, S, and VOM). This can be explained by the interaction between crop competitiveness and species-specific ecological traits. Legumes (except soybean) generally compete less effectively with weeds during the early growth stages, thereby creating more favorable conditions for weed emergence. In contrast, grain legumes and certain crop rotation sequences may have limited subsequent weed growth more effectively, resulting in lower weed biomass.

The differing responses among species indicate their ecological specialization: climbing species such as *L. purpureum* and *F. convolvulus* make better use of cereal crops following barley; *G. aparine* benefits from the taller and more open crop stands after soybean; while the early-emerging and shade-tolerant *V. arvensis* (Liu et al., 2023) competes successfully after pea and the vetch–oat mixture. Thus, weed distribution was determined both by the influence of the preceding crop on soil conditions and crop structure, and by the biological traits of the species.

### Undersown crops

In organic farming systems, udersown crops are an important tool for weed control. Cover crops are typically selected for their ability to emerge rapidly and grow vigorously, thereby covering the soil surface, limiting the availability of light, water, and nutrients to weeds, and promoting early weed germination followed by weed mortality due to competitive suppression. Undersown red clover can reduce weed density by up to 63.8%, while white clover can achieve reductions of up to 72.3% compared with pure cereal stands (Arlauskienė et al., 2021). White clover, due to its creeping growth habit, forms a dense ground cover, whereas annual Egyptian clover leaves inter-row spaces more open and creates more favorable conditions for weed establishment (Gecaitė et al., 2021). The effectiveness of undersowing was greatest in crops that were themselves less competitive with weeds (Arlauskienė et al., 2021). The inclusion of small proportions of aggressive plant species in mixtures can reliably enhance weed suppression (Baraibar et al., 2018). In addition, the undersowing of less common plant species (increasing weed diversity) may reduce overall weed abundance without negatively affecting crop productivity (Kimmel et al., 2025).

In our study, the highest weed emergence was recorded in the SB+RC–WW–SW rotation sequence, which is consistent with the literature indicating that undersown legumes can modify competition and soil coverage in ways that both suppress and, under some conditions, facilitate weed emergence. For example, the abundance of *G. aparine* and *V. arvensis* increased in spring wheat, while *F. convolvulus*, tolerant to competition, remained abundant even when emerging late (Špokienė and Povilionienė, 2003). These results highlight that the specific crop species, growth habit, and timing of undersown crops interact with weed traits to determine suppression effectiveness, supporting previous findings on the role of cover crops and spatial plant diversity in weed management (Isbell et al., 2017; MacLaren et al., 2020). In our study, the vetch–oat mixture showed weed suppression comparable to pure oat stands.

### Wheat competitiveness

Crop competitiveness is one of the key factors regulating weed proliferation. It can be enhanced using competitive crop species and cultivars, increased seeding rates, narrower row spacing, modified row orientation (Bajwa et al., 2017; Blank et al., 2023), mechanical or thermal reduction of weed density, and optimized nutrient management (Gallandt and Weiner, 2015). Winter cereals are generally more competitive than spring cereals due to their greater tillering capacity, faster early growth, and more effective soil surface coverage. Crop competitiveness is also strongly influenced by seeding rate and cultivar traits. This is supported by our results, which showed that total weed biomass in spring wheat was 4.3 times higher than in winter wheat. This supports findings from long-term rotation experiments showing that cereals with rapid early canopy development and high competitive ability contribute not only to reduce in-season weed biomass but also to long-term seedbank depletion (Gurusinghe et al., 2022).

Nitrogen availability further enhances crop competitiveness. Berquer et al. (2023) demonstrated that nitrogen-rich soils increase crop yield while reducing weed abundance.

Excess nitrogen availability or impaired growth of the main crops due to unfavorable conditions may stimulate weed proliferation, thereby requiring more intensive control. However, there is another nitrogen management strategy known as reverse fertilization. When plant residues with a high C:N ratio are incorporated, part of the soil nitrogen is immobilized in microbial biomass (nitrogen immobilization), thereby altering competitive interactions between crops and weeds (Gannett et al., 2024).

Many weed species are non–nitrogen-fixing plants and often have a lower root-to-shoot ratio, making them particularly sensitive to this strategy. As a result, their nitrogen supply is reduced and their competitive ability is weakened. Ecological modelling studies suggest that greater resource diversity can alter the competitive balance between crops and weeds, potentially favoring weed persistence (Bagavathiannan et al., 2020; Mwangi et al., 2024).

Our results showed that wheat competitiveness was enhanced by the inclusion of peas and soybeans in the crop rotation, as well as by the incorporation of red clover biomass. The highest weed biomass was recorded for *G. aparine* and *V. arvensis*. *F.-convolvulus* was the least suppressed species, consistent with previous findings indicating that its biomass may remain high even under well-established wheat stands (Riesinger and Hyvönen, 2006).

## Conclusions and insights

During the transition from conventional to organic farming, weed species richness increased by 23.3% on an average, with the most pronounced increase observed in crop sequences including soybean. The total weed abundance and the number of harmful weed species increased by 46.2% and 82.8%, respectively; however, the proportion of harmful weeds within the total weed community decreased compared with the beginning of the experiment.

Among legume pre-crops, the highest weed emergence was recorded following soybean crops, whereas the lowest occurred in spring barley undersown with red clover (RC). After grain legume pre-crops (peas, soybean, and the vetch–oat mixture), two consecutive years of winter and spring wheat cultivation increased arable weed emergence but reduced weed biomass. In contrast, the incorporation of nitrogen-rich red clover biomass increased both weed emergence and weed biomass in wheat.

These findings indicate that weed regulation was influenced less by legumes per se than by their residual (pre-crop) effects on subsequent crops. The strongest competitive pressure on cultivated crops was exerted by *G. aparine* and *F. convolvulus*, which produced the highest biomass per individual plant; particularly following legume pre-crops. The biomass of *V. arvensis* increased consistently throughout the study period. Legume pre-crops influenced both wheat growth and competitive interactions between weeds and crops.

Overall, crop rotation and undersown crops represent effective systemic strategies for weed management; however, their success depends on weed species composition, ecological traits, and the timing of control measures within the weed life cycle. Effective long-term control requires understanding weed biology, dispersal mechanisms, and the implementation of locally adapted management strategies.

## Data Availability

The original contributions presented in the study are included in the article/supplementary material. Further inquiries can be directed to the corresponding author.
